# Approach and assessment of automated stereotactic radiotherapy planning for early stage non-small-cell lung cancer

**DOI:** 10.1186/s12938-019-0721-7

**Published:** 2019-10-16

**Authors:** Xue Bai, Guoping Shan, Ming Chen, Binbing Wang

**Affiliations:** Department of Radiation Physics, Zhejiang Key Laboratory of Radiation Oncology, Zhejiang Cancer Hospital, Hangzhou, 310022 Zhejiang People’s Republic of China

**Keywords:** Machine learning, Non-small-cell lung cancer radiotherapy planning, Stereotactic body radiotherapy, Machine learning

## Abstract

**Background:**

Intensity-modulated radiotherapy (IMRT) and volumetric-modulated arc therapy (VMAT) are standard physical technologies of stereotactic body radiotherapy (SBRT) that are used for patients with non-small-cell lung cancer (NSCLC). The treatment plan quality depends on the experience of the planner and is limited by planning time. An automated planning process can save time and ensure a high-quality plan. This study aimed to introduce and demonstrate an automated planning procedure for SBRT for patients with NSCLC based on machine-learning algorithms. The automated planning was conducted in two steps: (1) determining patient-specific optimized beam orientations; (2) calculating the organs at risk (OAR) dose achievable for a given patient and setting these dosimetric parameters as optimization objectives. A model was developed using data of historical expertise plans based on support vector regression. The study cohort comprised patients with NSCLC who were treated using SBRT. A training cohort (*N* = 125) was used to calculate the beam orientations and dosimetric parameters for the lung as functions of the geometrical feature of each case. These plan–geometry relationships were used in a validation cohort (*N* = 30) to automatically establish the SBRT plan. The automatically generated plans were compared with clinical plans established by an experienced planner.

**Results:**

All 30 automated plans (100%) fulfilled the dose criteria for OARs and planning target volume (PTV) coverage, and were deemed acceptable according to evaluation by experienced radiation oncologists. An automated plan increased the mean maximum dose for ribs (31.6 ± 19.9 Gy vs. 36.6 ± 18.1 Gy, *P *< 0.05). The minimum, maximum, and mean dose; homogeneity index; conformation index to PTV; doses to other organs; and the total monitor units showed no significant differences between manual plans established by experts and automated plans (*P *> 0.05). The hands-on planning time was reduced from 40–60 min to 10–15 min.

**Conclusion:**

An automated planning method using machine learning was proposed for NSCLC SBRT. Validation results showed that the proposed method decreased planning time without compromising plan quality. Plans generated by this method were acceptable for clinical use.

## Background

Stereotactic body radiotherapy (SBRT) is an attractive alternative to lobar resection in patients with early stage non-small-cell lung cancer (NSCLC) not eligible for lobectomy. Compared with surgery, SBRT is noninvasive; it does not involve postoperative complications and can realize good local control [1, 2]. Intensity-modulated radiotherapy (IMRT) and volumetric-modulated arc radiotherapy (VMAT) are typical technologies used in NSCLC SBRT [3]. IMRT enables good conformity to tumor volume and low doses for healthy tissues [4]. Through the development of dynamic delivery using multi-leaf collimators (MLC), VMAT can deliver dose efficiently to reduce uncertainties in the intra-fraction setup [5, 6]. In IMRT, dose is delivered through a serial of static segments which is made up of modulated MLCs’ shapes. IMRT employs variable intensity across multiple radiation beams leading to the construction of highly conformal dose distributions. VMAT is an advanced IMRT technology [7]. The linac rotates around the patient and the MLCs continuously reshape and change the intensity of beams during dose delivery. Giving the radiotherapy in VMAT shortens the treatment time. VMAT is an alternative to fixed-gantry angle IMRT delivery. It allows the simultaneous variation of gantry rotation speed, treatment aperture shape via movement of MLC leaves, and dose rate during treatment delivery. One of the most important factors that affects the prognosis of IMRT and VMAT is the quality of the treatment plan, which depends on the experience and skill of the planner [8, 9]. Moreover, a plan is often developed through trial-and-error, necessitating a long planning time, which can be a limitation.

Several studies have focused on decreasing the interactions between the planner and the computer to reduce the planning time and improve the consistency of the plan quality. Methods such as template-based planning with the use of “scripting” tools [10–13], which imitate the trial-and-error process involved in manual planning, as well as multi-criteria optimization (MCO) [14–18] and knowledge-based treatment planning [19–26] have been investigated. Knowledge-based automated planning uses historical expertise plans as training data to learn the optimal strategies for new patients. This method has developed rapidly with the advancement of machine-learning algorithms. However, (1) only a few previous automated planning strategies have focused on SBRT, and (2) most existing studies have used the training data with an identical beam arrangement for different patients, such as seven or nine coplanar beams with equally spaced gantry angles in IMRT or a full-arc beam in VMAT [19–26]. These are listed in Table 1. Patient-specific beam arrangement has not been integrated in the knowledge-based automated planning studies.

**Table 1:** Studies on knowledge-based automated radiotherapy treatment plans

	Database	Site	Prediction method	Dosimetric parameter predictions	Beam angle predictions
Moore [19]	25 IMRT	Head-and-neck prostate	Analytical formulas	Mean doses of esophagus, larynx, parotid gland, bladder and rectum	No
Wu [20]	91 IMRT	Head-and-neck	Minimal dose approximation	Dose-volume objectives for head-and-neck organs	No
Zhu [21]	198 IMRT	Prostate	Machine learning	DVH of bladder and rectum	No
Petit [22]	25 IMRT	Pancreas	Minimal dose approximation	Dose-volume objectives for kidney and liver	No
Yang [24]	21 IMRT	Prostate	Linear regression	Rectal dose *D*_15_, *D*_20_, *D*_25_, *D*_35_, *D*_50_, and bladder dose *D*_15_	No
Nwankwo [25]	95 VMAT	Prostate	Machine learning	Uniformity index, *D*_10_, *D*_30_, *D*_50_, *D*_70_ and *D*_90_ in bladder and rectum	No
Wang [26]	80 IMRT	Esophagus	Machine learning	Mean heart dose and mean lung dose	No

Although identical beam arrangements are widely used in the traditional radiotherapy, customized partial arc radiation is generally better than an identical beam arrangement in an SBRT plan owing to variation in the tumor location and the low-dose radio sensitivity of healthy lung tissues. The shape, size, and position of tumors are important features that need to be considered for an optimized beam arrangement. Therefore, a strategy including either beam orientation optimization or available dose prediction is required to build automated SBRT planning tools.

Machine learning is a widely used method for analyzing big data from medical images and encapsulating it into task-specific information. Radiomics, medical image segmentation, and mass lesion classification based on machine-learning algorithms have brought about several breakthroughs in the diagnosis and treatment of cancer [27–31]. Machine learning-based automated planning systems have also been proposed by several studies [21, 25, 32, 33]. These methods have been proven efficient in head-and-neck cancer [34, 35] and prostate cancer [21, 32, 36]. In this study, the feasibility of a machine learning-based automated planning method was studied in NSCLC SBRT. We used a machine-learning method to determine the optimal strategy for an SBRT plan for lung cancer, including patient-specific objective function and beam arrangement. Anatomical features were extracted from individual patients, and the final beam arrangement and dosimetric results were predicted. Machine learning-based mathematical models were proposed and the feasibility of using this method in automated planning was validated. The objective of our study was to develop a fully automated NSCLC SBRT plan that aims to reduce planning time and meet the requirement for consistency in planning quality.

## Results

### Feature selection

The results of the feature selection are listed in Table 2. *V*_Heart_ and *Y*_PH_ were excluded for model building, because they had no significant correlation with beam and dosimetric features. *D*_PL_, *D*_PH_, *X*_PL_, *Y*_PL_, and *X*_PH_ were used in beam angle prediction, while *V*_PTV_, *V*_Lung_, *D*_PL_, *D*_PH_, OVZ_PL_, and OVZ_PH_ were used in dosimetric feature prediction.

**Table 2:** Spearman’s rank correlation test

	Lung dose			Beam angle	
	Mean	*V*_20_	*V*_10_	Start	Stop
*V*_PTV_					
*ρ*	0.747*	0.693*	0.731*	–0.033	–0.099
*P*	0.000	0.000	0.000	0.717	0.273
*V*_Lung_					
*ρ*	− 0.196*	− 0.205*	− 0.235*	0.073	0.021
*P*	0.029	0.022	0.008	0.416	0.813
*V*_Heart_					
*ρ*	0.014	0.005	0.023	− 0.077	-0.124
*P*	0.876	0.953	0.795	0.396	0.168
*D*_PL_					
*ρ*	− 0.508*	− 0.519*	− 0.467*	0.182*	0.234*
*P*	0.000	0.000	0.000	0.042	0.009
*D*_PH_					
*ρ*	− 0.241*	− 0.233*	− 0.203*	− 0.432*	− 0.412*
*P*	0.007	0.009	0.023	0.000	0.000
OVZ_PL_					
*ρ*	0.654*	0.639*	0.583*	− 0.33	− 0.66
*P*	0.000	0.000	0.000	0.713	0.466
OVZ_PH_					
*ρ*	0.385*	0.381*	0.254*	− 0.075	− 0.061
*P*	0.000	0.000	0.004	0.404	0.496
*X*_PL_					
*ρ*	− 0.098	− 0.154	− 0.075	0.762*	0.745*
*P*	0.277	0.087	0.405	0.000	0.000
*Y*_PL_					
*ρ*	− 0.100	− 0.155	− 0.074	0.758*	0.740*
*P*	0.265	0.085	0.415	0.000	0.000
*X*_PH_					
*ρ*	− 0.113	− 0.169	− 0.106	0.756*	0.729*
*P*	0.211	0.059	0.240	0.000	0.000
*Y*_PH_					
*ρ*	− 0.068	− 0.114	− 0.109	− 0.169	− 0.078
*P*	0.449	0.207	0.225	0.059	0.385

*Significant correlation

### Validation of the prediction model

**Fig. 1:**
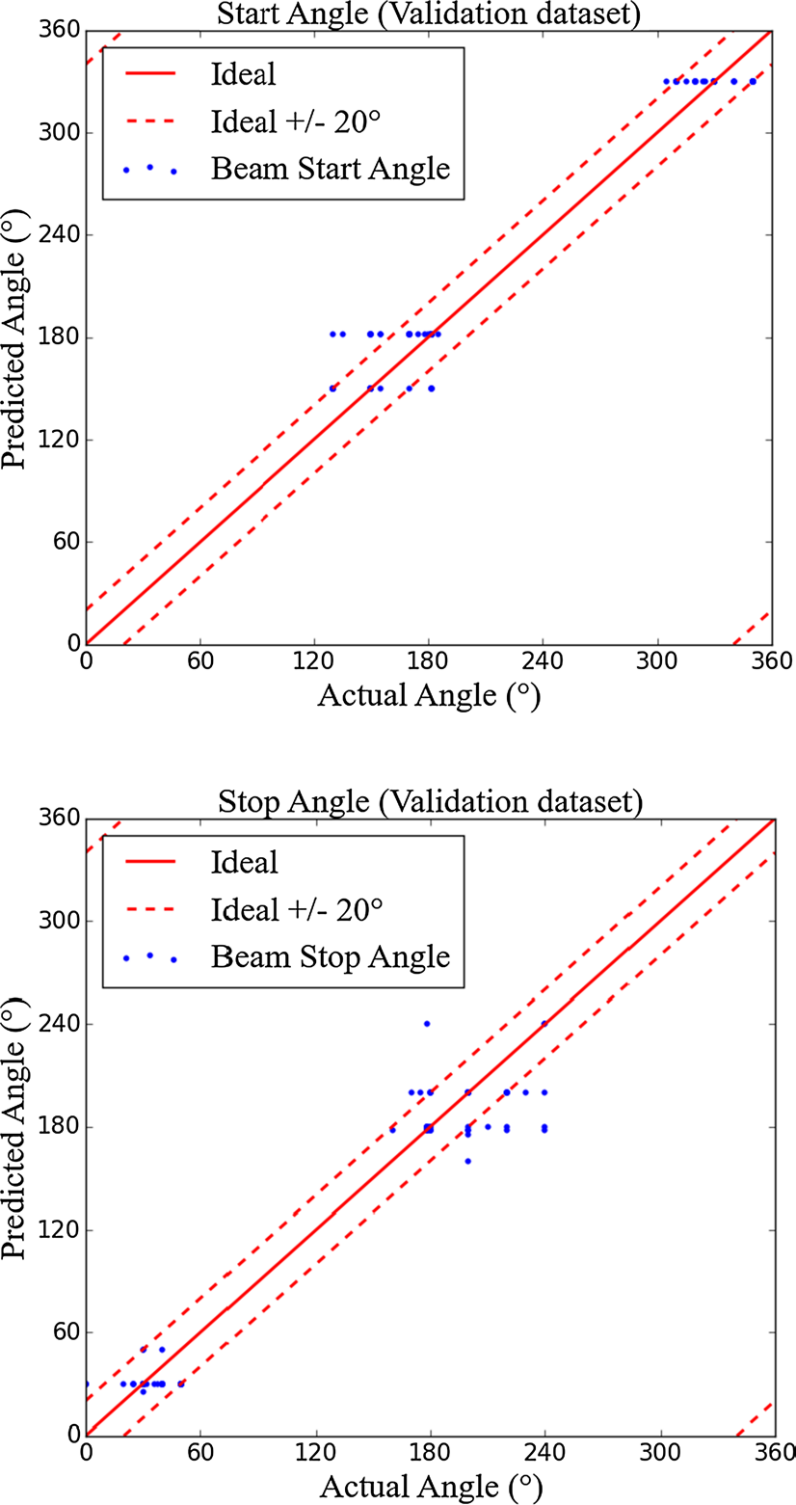
Prediction of the gantry angle in the training data sets for model validation

With regard to the CV of the LOO method, the root-mean-squared error (RMSE) of prediction for the start and stop gantry angles was 22.20° and 17.44°, respectively (Fig. 1). The RMSE prediction for the start and stop gantry angles for external validation data set was 18.5° and 9.6°, respectively.

**Fig. 2:**
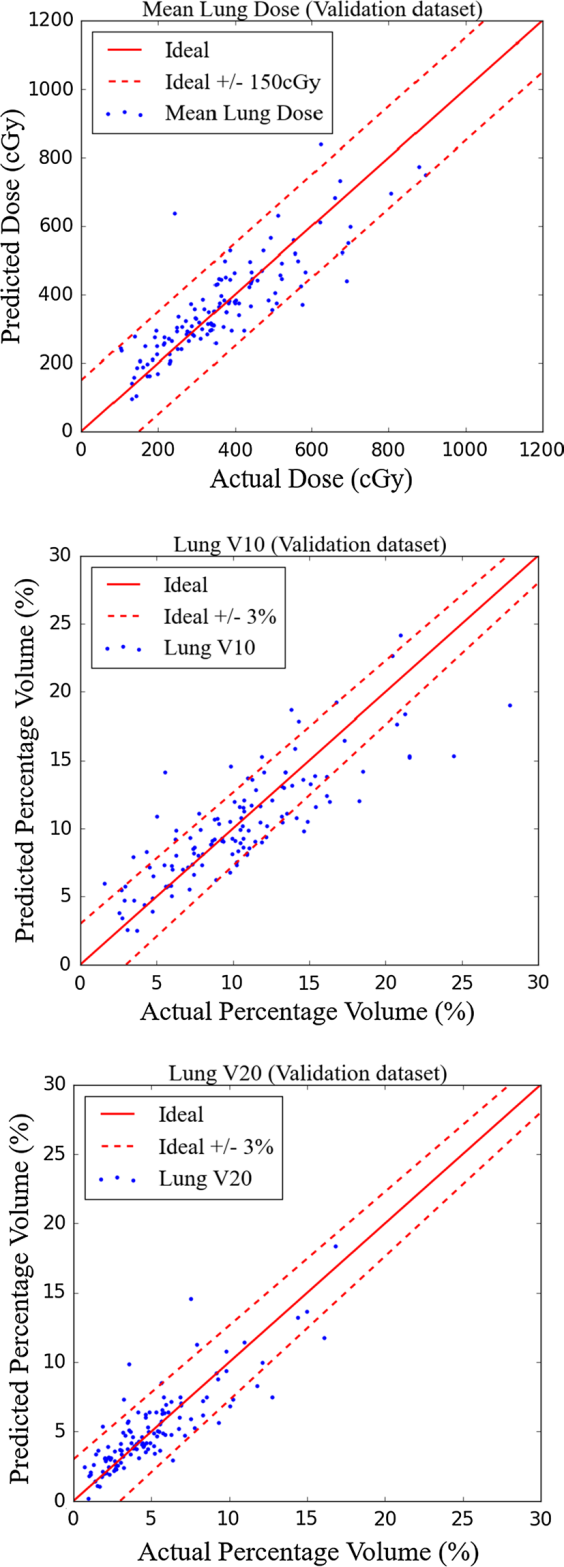
Dose prediction in the training data sets for model validation

The RMSE of the predicted mean lung dose (MLD), *V*_10_, and *V*_20_ of the lung was 0.83 Gy, 3.10%, and 1.71%, respectively, for CV (Fig. 2). For external validation, the RMSE of the predicted MLD, *V*_10_, and *V*_20_ of the lung was 1.00 Gy, 3.64%, and 2.40%, respectively.

### Comparison of the quality of plans

**Fig. 3:**
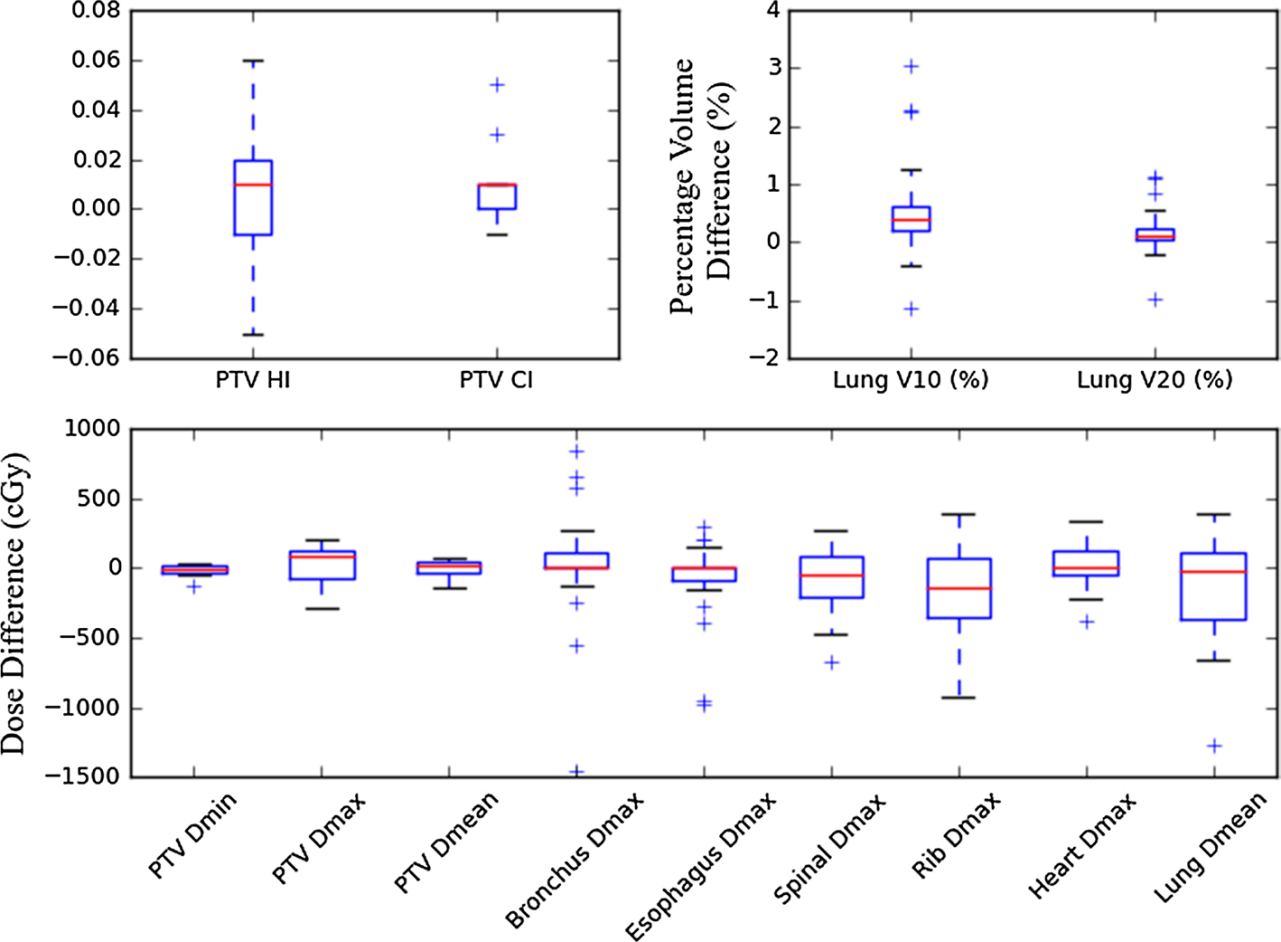
Boxplot showing the dose difference between the manual and automated plans for each ROI. Positive values represent reduced dose in the automated plan and vice versa

Table 3 shows a comparison of all 60 plans for 30 patients in the testing set. According to the radiation oncologists, 30/30 automated plans fulfilled the dose criteria for OARs and PTV coverage for Zhejiang Cancer Hospital and were acceptable for clinical treatment. A significant difference was observed only in the mean *D*_max_ of the rib: 31.6 ± 19.9 Gy in the manual plan and 36.6 ± 18.1 Gy in the automated plan (*P *< 0.05). Doses to other organs and PTV, as well as the total monitor units, showed no significant differences between the two planning strategies (*P *> 0.05). Figure 3 shows a boxplot of dose differences between the manual plan and the automated plan for each ROI.

**Table 3:** Clinical constraints and dose results for various indices

Index	Constraints	Manual plan		Automated plan		*P* value
		Mean	SD	Mean	SD	
PTV *D*_min_ (Gy)	> 45.5	48.4	0.5	48.4	0.3	0.837
PTV *D*_max_ (Gy)	< 70.0	66.7	2.5	69.8	2.1	0.594
PTV *D*_mean_ (Gy)	–	58.1	1.3	58.5	1.3	0.515
PTV HI	–	0.31	0.04	0.32	0.03	0.478
PTV CI	> 0.8	0.87	0.04	0.88	0.03	0.157
Bronchus *D*_max_ (Gy)	< 30.0	9.3	12.5	9.8	10.7	0.841
Esophagus *D*_max_ (Gy)	< 32.5	5.9	6.0	6.5	5.8	0.472
Spinal *D*_max_ (Gy)	< 30.0	9.7	5.1	8.7	4.2	0.082
Rib *D*_max_ (Gy)	< 54.0	31.6	19.9	36.6	18.1	0.005
Heart *D*_max_ (Gy)	< 30.0	13.1	12.2	13.0	11.6	0.658
Lung *D*_mean_ (Gy)	< 5.0	3.4	1.4	3.3	1.4	0.082
Lung *V*_10_ (%)	< 15.0	9.7	4.3	9.2	4.1	0.050
Lung *V*_20_ (%)	< 10.0	4.6	2.6	4.4	2.5	0.658
Total MU	–	1078	217	1054	198	0.594

The hands-on planning time was estimated and could be reduced from approximately 40–60 min to 10–15 min using the automated planning procedure.

## Discussion

This study demonstrates that machine-learning method can be used to generate clinically acceptable treatment plans automatically for NSCLC SBRT patients. All the plans were evaluated and recognized by two radiation oncologists. The anatomical geometry features of patients were extracted from 125 CT scans. The SVR was used to find the relationship between these features and the optimized plan parameters for training plans. Thereafter, the regressive models were used to generate radiotherapy plans automatically. As shown in Table 3, for all the 30 validation patients, the automated plans generated were comparable with the plans created by expert planners, and were acceptable for clinical treatment.

In this study, the SVR was used to fit geometry features and plan parameters instead of the classical regression method, because the former has the advantage of preventing over-fit via stopping penalty when the error between predicted and actual values is less than a given value. Zhu [21] implemented SVR to establish the correlation between the features of the DVH and the anatomical information in a database consisting of 198 high-quality prostate plans. SVR can manage over-fit when the data size increases. Furthermore, the best plan parameters were not identical owing to the subjective variations introduced by the planners. An expert plan could be achieved by setting the parameters in different ways, while its robust quality could be attained through minor changes in parameters. The error tolerance of SVR was appropriate in the prediction of plan parameters.

Although beam orientation optimization is essential and widely discussed [37–39], previous studies regarding automated plans often used full-arc beam arrangements rather than an optimized one. One of the reasons for this is that PTV with an irregular shape is more difficult for beam orientation prediction [26]. However, because of the variations in tumor position and low-dose sensitivity of healthy lung tissues, optimized beam arrangement may potentially reduce the complications in normal tissue. In that case, the beam arrangement strategy must be different from previous auto plan studies which focus on either head-and-neck [20, 23] or pelvic [24, 25, 40] regions. Data set involved in this study includes convex PTVs in early stages of NSCLCs, making the prediction of beam orientation feasible. The auto-beam arrangement based on anatomy was proposed and validated in this study. *iCycle* used a priori multi-criteria approach to combine beam angle choice into automated plan by sequentially adding beams in an iterative procedure [16]. Our SVR method could attain the optimized beam arrangement in a single step.

Feature selection in this study is different from that reported in previously published studies that employed overlap-volume histogram (OVH) to infer the dose-volume levels [20, 23, 24, 41]. The OVH represents percentage volumes of an OAR that is within a specified distance of PTV boundary. The OVH fails to describe the influence of space coordinates in the voxel dose. Moreover, a greater number of features increase the accuracy of the model but also increase the complexity of modeling and the risk of over-fitting, requiring a large number of training examples for model construction. Besides the features commonly used in dose prediction, such as ROI volumes and distances between PTV and OARs, three-dimensional coordinate information for each OAR was used to describe their relative position in the present study. Using this method of choosing anatomy factors, the calculated beam angle and predicted OAR dose can be obtained in real time.

The purpose of this study was developing an automated planning procedure for VMAT plans. IMRT plans were also enrolled to enlarge the size of training data set. Because for most tumor site, VMAT and fixed IMRT could produce largely equivalent target volume coverage, dose conformity, and homogeneity [42, 43], using a hybrid training data set is reasonable. Although there were 59.2% IMRT plans (74 in 125) in training data set, while the test data sets were all VMAT plans, the prediction results were acceptable for the test data set. The result proves that static IMRT data could be used for training VMAT models.

In this study, predictions of only three parameters, *V*_10_, *V*_20_, and *D*_mean_ of the lung, were made to ensure that the procedure for the automated treatment plan remained uncomplicated. Since the expected benefit from further reducing the *D*_max_ of serial organs compared with the constraint value is limited and not aimed for clinical practice, the optimization objectives for the heart, bronchus, spine, esophagus, and rib were programmed as a template and kept constant for all patients in the study. The key dosimetric difference between the automated treatment plan and manual plan in this study was the maximum dose for the ribs. This was because a number of patients for whom the distance between the PTV mass center and the ribs mass center was short were enrolled. The aim of future studies would be to predict the doses for serial organs and combine the results into an objective function.

Our study is novel in that it calculated the best start and stop angles for VMAT using a machine-learning method, which can be a valuable supplement for the automated VMAT plan. The geometrical features used to build the prediction model could be readily extracted from CT scans using the scripts tool in RayStation, enabling the automated tool to be applicable to regular practice. In theory, the study proved the relationship between anatomy geometry and the treatment plan parameters for NSCLC SBRT. Based on this relationship, an automated planning tool was developed to reduce planning time and meet the requirements for plan quality.

There are several limitations to this study. First, only coplanar beam plans with 0° collimator were considered for the training data set. Studies on SBRT considering advanced models with non-coplanar fields and patient-specific collimator angle will be conducted in the future. Second, although comparison of the automated and manual plans revealed that the former could achieve acceptable sparing of critical structures for the 30 patients in the test data set, the results could not prove that those plans are the most optimal ones in the present study. Other optimization parameters, such as objective weight, gantry spacing, and template-based objective functions, must also be studied to determine optimal values. This work is a foundation for further study, and better training data sets should be used to produce better quality plans in the future.

## Conclusions

Using features of the anatomy of patients to generate a predicted arc angle and an objective function for an automated treatment plan is feasible. The procedure for an automated treatment plan was developed in this study involving set start and stop angles for the beam arc as well as objective functions that would operate without manual intervention. The dosimetric impact of the automated plan on the PTV, bronchus, esophagus, spinal cord, heart, and lung was insignificant (*P *< 0.05). In the automated plan, the mean maximum dose for the ribs was increased (31.6 ± 19.9 Gy vs. 36.6 ± 18.1 Gy, *P *< 0.05). All the 30 validation cases produced results acceptable for applying this method to clinical treatments based on oncologists’ evaluation. This procedure could reduce the planning time by nearly three-quarters of the time required to formulate manual plans while generating plans that are as good as ones designed by an experienced planner. Therefore, the plans generated by this method are acceptable for clinical use.

## Methods

### Patient data

A total of 125 patients with stage-I NSCLC underwent SBRT in 2015–2017 according to the protocols of the Zhejiang Cancer Hospital, Zhejiang, China. Clinical treatment plans for all patients were generated using the RayStation TPS (RaySearch Laboratories, Stockholm, Sweden).

The clinical target volume, planning target volume (PTV), and organs at risk (OARs) were delineated by experienced radiation oncologists and reviewed by senior physicians. The data comprised 74 IMRT plans and 51 VMAT plans. The IMRT plans were delivered using 11–13 step-and-shoot coplanar beams with a gantry spacing of 20° between the beams and arranged in a fan shape; the plans had dosimetric features similar to those of the VMAT plans. The latter were delivered using two arcs with a gantry spacing of 4° between the control points, with the distance between the start and stop angles varying from 220° to 260°. The start and stop angles of the arcs were decided by expert planners based on the anatomy of individual patients. The PTV was 3.19–357.20 cm^3^ (mean, 36.92 cm^3^). Patients were treated using five fractions and prescribed 50 Gy to the PTV. The prescription dose covered at 95% of the PTV, and the maximum dose did not exceed 150% of the prescription dose. The dosimetric constraints of the OAR partly consulted Radiation Therapy Oncology Group (RTOG) protocols 0813 and 0915, and are listed in Table 3. To conform the ALARA principle, all plans were optimized further using a trial-and-error process to achieve optimal sparing of OARs and were considered expert plans. These plans were used for clinical treatments and for the present study.

### Characteristics of plans: geometry features, beam angles, and achievable dose for organs at risk (OARs)

In radiotherapy, the parameters of treatment plans are determined by the planners according to anatomical data based on computed tomography (CT) images. Intuitively, the beam orientation and constraints of OAR dose tend to correlate with the anatomic features of images from patients.

**Fig. 4:**
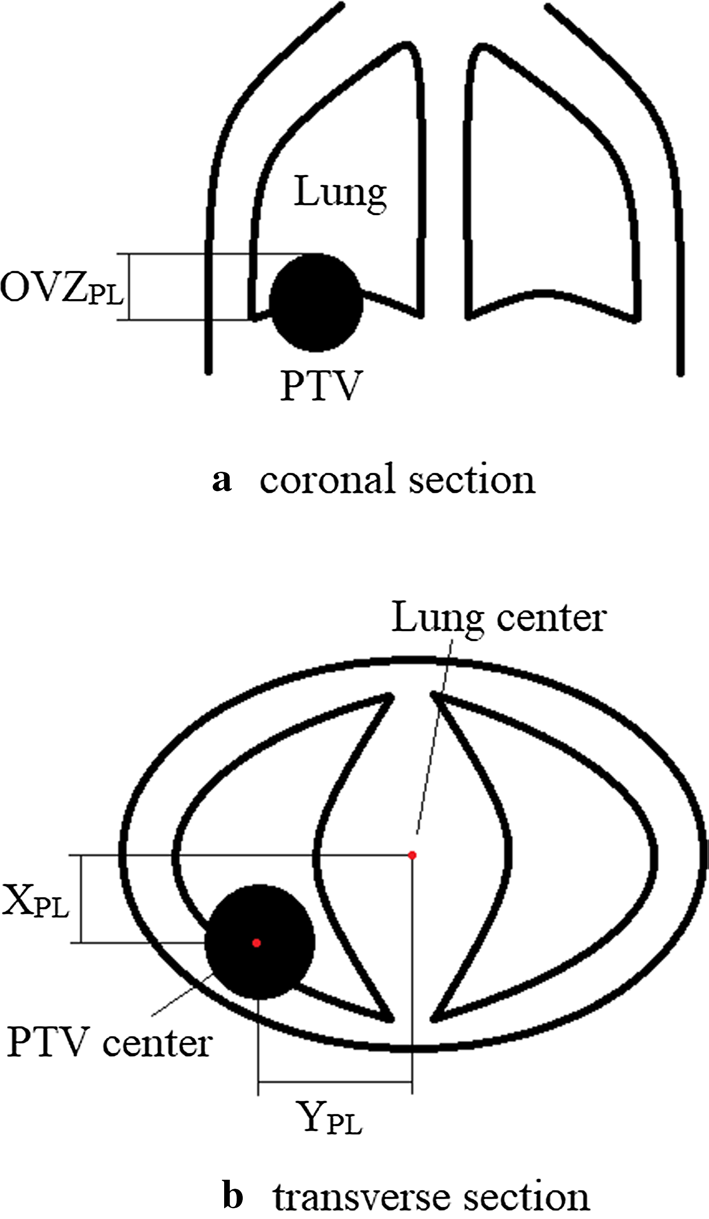
OVZ_PL_, *X*_PL_, and *Y*_PL_ demonstration

In the present study, 11 anatomical features were extracted from digital imaging and communications in medicine documents: (1) PTV volume (*V*_PTV_); (2) lung volume (*V*_Lung_); (3) heart volume (*V*_Heart_); (4) distance between the PTV mass center and the lung-mass center (*D*_PL_); (5) distance between the PTV mass center and the heart-mass center (*D*_PH_); (6) overlap length of the PTV and the lung in the *z*-axis (OVZ_PL_, introduced by Wang et al. to predict the Pareto front in esophageal cancer [26]); (7) overlap length of the PTV and the heart in the *z*-axis (OVZ_PH_,); (8) distance between the PTV mass center and the lung-mass center in the *x*-axis (*X*_PL_); (9) distance between the PTV mass center and the lung-mass center in the *y*-axis (*Y*_PL_); (10) distance between the PTV mass center and the heart-mass center in the *x*-axis (*X*_PH_); and (11) distance between the PTV mass center and the heart-mass center in the *y*-axis (*Y*_PH_). PTV is major concerned in treatment planning, in that 9 PTV related features were extracted. Meanwhile, delivered tumor cause inevitable dose to lung and heart which may cause radiation toxicity in normal tissue. Five lung-related features and five heart-related features were also extracted to evaluate delivery dose in this study. The OVZ_PL_, *X*_PL_, and *Y*_PL_ are shown in Fig. 4. These data could describe the volume, relative position, and shape of the regions of interest (ROIs), because the tissue anatomy of each patient was similar. The start and end angles of the arc or IMRT fan were recorded as features of the beam angle. The couch and collimator angles were 0° for all cases. The *V*_10_ (percentage lung volume of 10 Gy) and *V*_20_ (percentage lung volume of 20 Gy) of the lung and mean lung dose were recorded to represent the dose features. These dosimetric parameters were exported from the TPS using Python scripts.

### Feature selection

Spearman’s rank correlation test was used to evaluate the correlation between the anatomical features and the beam angle and dosimetric features. Spearman’s rank correlation coefficient is a non-parametric rank statistic proposed as a measure of the strength of the association between two variables. It can be used in feature selection without making any assumptions about the frequency distribution of the variables [26]. If the *P* value was > 0.05, no significant correlation was found between the two variables. Irrelevant anatomical features were excluded from prediction modeling. The reserved features were used to predict beam angle and lung dose before determining the beam angle and objective function parameters for an automated plan using a machine-learning model.

### Prediction and validation

**Fig. 5:**
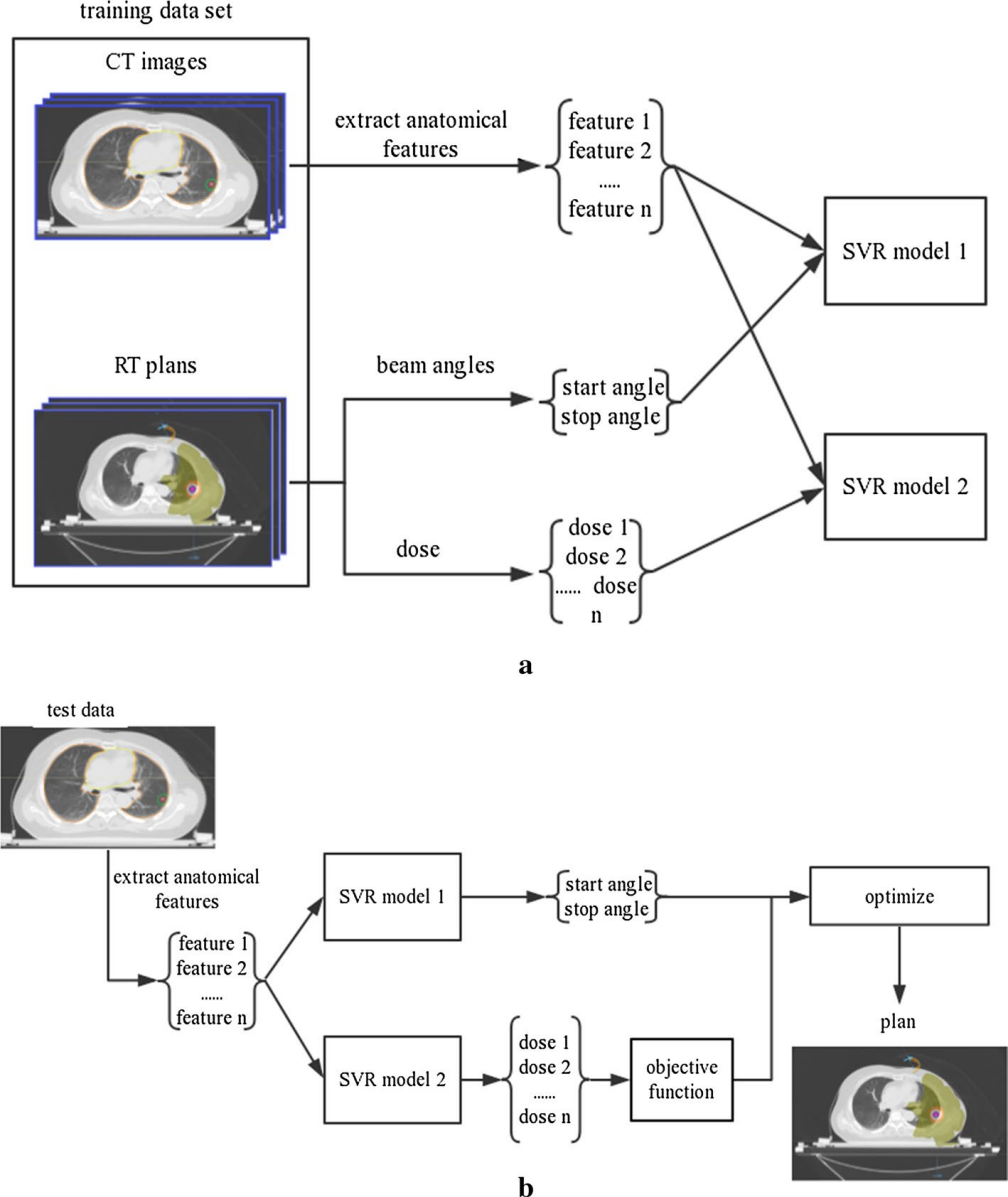
Flowchart of the major steps in automated planning: **a** Modeling method. **b** Use of the two models

Figure 5 is a flowchart of the major steps in the automated planning. The goal of training is to establish two mathematic correlations. One maps the anatomic information extracted from patient images and the selection of the beam angle. The other maps the anatomic information and *V*_10_, *V*_20_, and the mean dose of the lung. For convenience in the modeling, all plans were normalized at 95% of PTV in 50 Gy. All training data were standardized by removing the mean values and scaling to unit variance as a common requirement for machine-learning estimators.

Support vector regression (SVR) was implemented as the modeling method. SVR is a supervised learning method used for data regression. For complicated problems that are not regressed to a linear function, SVR introduces a kernel function that projects the data into a higher dimensional space where it can be regressed to a linear function. By introducing the kernel, SVR gains flexibility in the choice of the form of regression function, which needs not be linear and even needs not have the same functional form for all data, since its function is non-parametric and operates locally. As a consequence, they can work with geometry features which show a non- linearly relation to the beam angles and OAR doses. SVR also introduces a tube of width *ε*; and finding a function that is at most *ε* deviations from the targets actually obtained for all the training data becomes problematic [44, 45]. By choosing an appropriate *ε*, SVR can be robust even when the training data have some bias; for example, the results of training plans are slightly variation according to the discretion of planers. In machine learning, a hyperparameter is a parameter whose value is set before the learning process begins. Hyperparameters include *ε* and the kernel function, which are not learned directly within the estimators in SVR. An exhaustive grid-search method was introduced to find the appropriate values for the two hyperparameters, by searching the parameter space for the best cross-validation (CV) score. The CV method is used to determine how the results of statistical analysis generalize to an independent data set. The leave-one-out (LOO) method was used in each model for CV. In LOO, an entire data set with *n* patients was separated into a training data set with *n* − 1 patients and a validation data set with 1 patient [46]. The SVR model was developed using the training data set and applied to the validation data set. The SVR and Gridsearch algorithms were implemented using Scikit-learn [47].

A total of 125 cases were used in the training data set. With an LOO method, in each iteration, one case was randomly chosen as validation set and other 124 cases were training sets for cross validation. After models training, 30 cases outside the training pool were used as a test set for external validation. The actual values of the gantry start angle and stop angles, *V*_10_, *V*_20_, and mean dose of the lung were collected from the treatment plans generated by expert planners. The corresponding predicted value was calculated using a prediction model and the standard deviation of the resulting error was calculated.

### Automated planning approach and assessment

Two factors of planning were determined automatically: gantry angles and objective functions. The start and stop gantry angles were predicted and used as an arc parameter for VMAT. Objective function parameters were calculated from the machine-learning model and individualized. For each patient, an automated plan was generated based on the predicted arc start and stop angles and the optimization objectives.

As a test for the automated planning procedure, two strategies were used to develop SBRT plans for the 30 cases in the testing set: (1) manual plan (designed by an experienced planner through trial-and-error) and (2) automated plan (designed by the automated planning procedure). All 60 plans were normalized at a 50-Gy dose (for 5 fractions) covering 95% of the PTV.

Two experienced radiation oncologists at Zhejiang Cancer Hospital reviewed the dose–volume histograms (DVHs) and dose distributions of the 30 automated plans and judged the acceptability of the plans for clinical treatments. The radiation oncologists was asked to decide each plan was clinical acceptable or not. For the PTV, the mean dose (*D*_mean_), maximum dose (*D*_max_), minimum dose (*D*_min_), and homogeneity index (HI) as defined by the International Commission on Radiation Units and Measurements 83 [48], and the conformity index (CI) as defined by Paddick et al. [49] were evaluated. For OARs, *D*_max_ for the bronchus, esophagus, spine, ribs, and heart, as well as *D*_mean_, *V*_10_, and *V*_20_ for the lung, were evaluated for comparison.

### Statistical analyses

Statistical analyses of dosimetric differences were performed using the Wilcoxon rank test based on the correlation between the manual plan and the automated plan using SPSS v21 (IBM, NY, USA). A *P* value < 0.05 was considered significant.

## Data Availability

The data sets used and/or analyzed during the current study are available from the corresponding author on reasonable request.

## Abbreviations

IMRT: intensity-modulated radiotherapy
VMAT: volumetric-modulated arc therapy
SBRT: stereotactic body radiotherapy
NSCLC: non-small-cell lung cancer
OAR: organs at risk
PTV: planning target volume
MCO: multi-criteria optimization
CT: computed tomography
RTOG: radiation therapy oncology group
CI: conformity index
SVR: support vector regression
CV: cross validation
LOO: leave one out
OVH: overlap-volume histogram
RMSE: root-mean-squared error
MLD: mean lung dose
